# Involvement of CD244 in Regulating CD4^+^ T Cell Immunity in Patients with Active Tuberculosis

**DOI:** 10.1371/journal.pone.0063261

**Published:** 2013-04-30

**Authors:** Bingfen Yang, Xinjing Wang, Jing Jiang, Xiaoxing Cheng

**Affiliations:** Division of Research, Institute of Tuberculosis, 309 Hospital, Beijing, China; St. Jude Children's Research Hospital, United States of America

## Abstract

CD244 (2B4) is a member of the signaling lymphocyte activation molecule (SLAM) family of immune cell receptors and it plays an important role in modulating NK cell and CD8^+^ T cell immunity. In this study, we investigated the expression and function of CD244/2B4 on CD4^+^ T cells from active TB patients and latent infection individuals. Active TB patients had significantly elevated CD244/2B4 expression on *M. tuberculosis* antigen-specific CD4^+^ T cells compared with latent infection individuals. The frequencies of CD244/2B4-expressing antigen-specific CD4^+^ T cells were significantly higher in retreatment active TB patients than in new active TB patients. Compared with CD244/2B4-dull and -middle CD4^+^ T cells, CD244/2B4-bright CD4^+^ T cell subset had significantly reduced expression of IFN-γ, suggesting that CD244/2B4 expression may modulate IFN-γ production in *M. tuberculosis* antigen-responsive CD4^+^ T cells. Activation of CD244/2B4 signaling by cross-linking led to significantly decreased production of IFN-γ. Blockage of CD244/2B4 signaling pathway of T cells from patients with active TB resulted in significantly increased production of IFN-γ, compared with isotype antibody control. In conclusion, CD244/2B4 signaling pathway has an inhibitory role on *M. tuberculosis* antigen-specific CD4^+^ T cell function.

## Introduction

Tuberculosis (TB) is the second leading cause of death from an infectious disease worldwide [1]. It is estimated that 8.8 million cases of TB occurred in 2010 and 2.6 million were smear-positive. In 2010 alone, there were estimated 1.1 million deaths from TB in HIV-negative people and 0.35 million deaths from HIV-associated TB [1].

Despite high rate of *Mycobacterium tuberculosis* infection in humans, especially in developing countries, only 5–10% of infected people develop into active TB in their life time [2], [3], [4]. The observation suggests that development into active TB is largely determined by immune responses of the host. Previous studies have proved the critical role of CD4^+^ T cells in protective immunity against *M. tuberculosis* infection, while other cells, such as CD8^+^ T cells, γδ T cells and CD1-restricted T cells also play important roles [2], [5], [6], [7], [8], [9], [10]. CD4 knockout mice demonstrated increased susceptibility to *M. tuberculosis* infection, compared with wild-type mice [5]. AIDS patients have severe defects in CD4^+^ T cells and are highly susceptible to development of active TB [1], [6].

T cell immune responses are regulated by different activating and inhibitory surface receptors. *M. tuberculosis* infection promotes up-regulation of inhibitory receptor PD-1, its ligands, PD-L1 and PD-L2, on T cells from patients with active TB. Blockage of PD-1 or PD-1 and its ligands leads to significantly improved IFN-γ production and degranulation of T cells [11]. PD-1^−/−^ mice have excessive inflammatory responses after *M. tuberculosis* infection [12]. The T cell immunoglobulin and mucin domain-containing molecule 3 (Tim-3), an inhibitory receptor highly expressed on exhausting T cells [13], is up-regulated on both total CD8 and antigen-specific CD8 T cells from active TB patients [14]. The elevated expression of Tim-3 on CD8 T cells is significantly associated with T cell dysfunctions and disease severity of TB patients. Blocking of Tim-3 signaling led to significantly increased production of IFN-γ [14]. These studies indicate that the PD-1 and Tim-3 signaling pathways inhibit T cell effector functions during *M. tuberculosis* infection. It would be interesting to investigate whether other costimulatory receptors are involved in the regulation of anti-TB immunity.

CD244 (also called 2B4) is a member of the signaling lymphocyte activation molecule (SLAM) family of immune cell receptors [15], [16], [17]. It is expressed on natural killer (NK) cells, CD4 and CD8 T cells, γδ T cells, monocytes, eosinophils and basophiles [18]. The function of CD244/2B4 on NK cells has been studied extensively; it was initially described as an activating receptor and was later found to have both activating and inhibitory functions in mouse NK cells [15], [16], [19], [20], [21]. The phosphorylated ITSMs of CD244/2B4 tail can bind to signaling lymphocyte activation molecule–associated protein (SAP), and it also can recruit phosphatases such as SHP-1, SHP-2, SHIP, and the inhibitory kinase Csk [21]. It is found that 2B4 exhibited an activating function when expressed at low levels, while generated an inhibitory signal when expressed at high levels [22].

Previously studies have found that CD244/2B4 plays an important role in modulating CD8^+^ T cell immunity during infection [23], [24], [25], [26], [27]. To our knowledge, the role of CD244/2B4 on human CD4^+^ T cell function in TB patients has not been reported so far. CD4 T cells play a central role in human immune protection and its importance is clearly demonstrated in AIDS patients. Depletion in CD4 T cells leads to systematic infection and cancer. Since CD4 T cells have critical role in anti-TB immunity, it would be interesting to study whether CD244/2B4 has any influence on function of CD4 T cells during active disease of TB.

In this study, we investigated the expression and function of CD244/2B4 on CD4^+^ T cells from active TB patients, latent infection individuals and healthy controls. We demonstrated that CD244/2B4 is involved in regulation of CD4^+^ T cells immunity in patients with active TB.

## Materials and Methods

### Ethics Statement

The study was approved by the Ethics Committee of Beijing 309 Hospital, and informed written consent was obtained from all participants.

### Human subjects

Fifty-two patients diagnosed as active TB were recruited from clinics of the Institute of Tuberculosis, 309 Hospital, Beijing, China, during period of March 2011 to December 2011. Twenty-six patients were sputum smear- and/or culture-positive and 26 were negative (Table 1). For patients with negative bacterial culture results, diagnosis was established by chest radiography, clinical symptoms and pathological examination from biopsy samples obtained through bronchoscopy, and was confirmed by symptomatic and radiographic improvement after anti-TB chemotherapy. Individuals with malignant tumor, HIV infections were excluded.

**Table 1:** Demographic and clinical characteristics of TB patients, latent infection individuals and healthy controls.

	Active TB patients	Latent TB infection	Healthy controls
Sex N(female/male)	52 (30/22)	12 (7/5)	38 (17/21)
Age(mean±SD)	34.88±1.56	34.17±3.15	33.95±1.346
ELISPOT-positive	23/32	12/12	0/38
*M. Tuberculosis* culture-positive	26/52	ND^a^	ND
Lung cavity	20/52	0/12	0/38
New/retreatment/others^b^	30/13/9	N/A^c^	N/A

Note: ^a^ ND: not determined. ^b^ New/retreatment: new or retreatment active TB patients; others: TB patients with 1–5 month treatment duration. ^c^ N/A: not applicable.

In the cohort of active TB patients recruited in the study, 30 were defined as new cases and 13 as retreatment patients. TB patients who were not treated for TB or were treated for less than one month at time of blood collection were considered new cases. Thirteen TB patients who were previously treated with standard anti-TB chemotherapy regimen without success were admitted to the hospital for retreatment, and this group of patients was defined as retreatment group.

Healthy controls were randomly recruited from individuals undergoing annual health check-up at the clinics of 309 Hospital, with following inclusion criteria: (1) no fever, cough or other signs of active TB; (2) with normal physical examination result and normal radiography; (3) without HIV infection. Latent infection individuals were defined as T-SPOT.TB-positive (Table 1). This study was approved by the Ethics Committee of the 309 Hospital and informed consent was obtained from all subjects.

### Preparation of human PBMCs

Fresh peripheral whole blood obtained from TB patients and control individuals was treated with heparin to prevent coagulation. Peripheral blood mononuclear cells (PBMCs) were purified by density gradient centrifugation using Ficoll-Paque PLUS (GE Biosciences, USA). 1×10^6^ PBMCs were washed twice with RPMI 1640 (GIBCO, New York, USA) respectively, and then resuspended in AIM V® Serum Free Medium. The cells were added to 96 well tissue culture plate (Corning, New York, USA), 200 ml/well.

### Surface antibody staining and flow cytometric analysis

PBMCs were incubated overnight at 37°C in CO_2_ incubator with peptide pools encompassing *M. tuberculosis* antigens ESAT-6 and CFP-10 for 16 h. Each individual peptide in the pools was at a final concentration of 1 μg/ml. After incubation, all cells were collected, washed, stained with anti-CD244-PE, anti-CD69-FITC (BD Biosciences, San Diego, California, USA), anti-CD3 PE-Cy5 and anti-CD4 PE-Cy7 (BioLegend Inc., San Diego, CA, USA), and analyzed by an FC-500 flow cytometer (Beckman Coulter, Brea, CA, USA).

### Intracellular cytokine staining

PBMCs from patients with active pulmonary TB were stimulated with heat-inactivated *M. tuberculosis* strain H37Rv lysates at a final concentration of 10 μg/ml, as estimated by BCA assay. Brefeldin A (Sigma-Aldrich, St. Louis, MO, USA) was added one hour after antigen stimulation. After 16 hr of incubation, cells were collected, washed, stained with surface markers. After permeabilized, the cells were incubated with anti-IFN-γ-FITC or IgG2b-FITC isotype antibody control (R&D Systems, Minneapolis, MN, USA) for 30 minutes at 4°C. The production of cytokines was analyzed by FC-500 flow cytometer.

### IFN-γ-release assay

IFN-γ-release assay was performed by using T-SPOT.TB kit (Oxford Immunotec, Oxfordshire, United Kingdom) by following manufacture's instruction. Briefly, PBMCs obtained by active TB patients and healthy controls were added to 96-well plates, 2.5×10^5^ cells/well, and were stimulated with *M. tuberculosis* antigen ESAT-6 and CFP-10 peptide pools for 20 h at 37°C. Spots were counted by using CTL-ImmunoSpot® Analyzer (Cellular Technology Ltd, Shaker Heights, OH, USA).

### Cross-linking or blocking of CD244/2B4 signaling pathway with antibodies

PBMCs from 24 patients with pulmonary TB were stimulated with *M. tuberculosis* ESAT-6 and CFP-10 overlapping peptides in the presence of activating anti-2B4 antibody clone C1.7 (functional grade), or blocking anti-2B4 antibody clone eBioPP35 (functional grade), or purified mouse IgG1 isotype controls (functional grade) (all from eBioscience, San Diego, CA, USA) at a concentration of 10 μg/ml. For flow cytometric analysis, Brefeldin A (Sigma-Aldrich, St. Louis, MO, USA) were added to the culture medium to a final concentration of 1 µg/ml one hour later. IFN-γ was detected by intracellular cytokine staining and flow cytometric analysis after 6 h in vitro stimulation, or by ELISPOT assay by using both ESAT-6 and CFP-10 peptide pools as stimulators.

### Statistical analysis

Mann-Whitney and paired t-test analysis were used for statistical analysis between groups by using GraphPad Prism version 5.01 (GraphPad Software, San Diego, CA, USA). Data were shown as mean ± standard derivation (SD). All tests were two-tailed and p<0.05 was considered significant.

## Results

### Clinical characteristics of TB patients, latent infection individuals and healthy controls

Fifty-two patients with active TB and 50 healthy controls were included in the study. All of healthy control individuals were screened by an ELISPOT assay called T-SPOT.TB which uses *M. tuberculosis* antigens ESAT-6 and CFP-10 peptide pools as stimulators, and 12 of them were positive (24%) and were recruited as latent TB infection individuals. The demographic and clinical characteristics of all TB patients, latent infection individuals and healthy controls were listed in table 1. Among the 52 active TB patients, 32 were tested by T-SPOT.TB assay and 23 were positive (71.9%). The relative low positive rate of T-SPOT.TB in TB patients was in consistent with previous reports [28].

### Expression of CD244/2B4 on CD4^+^ T cells in active TB patients, latent infection individuals and healthy controls

**Figure 1:**
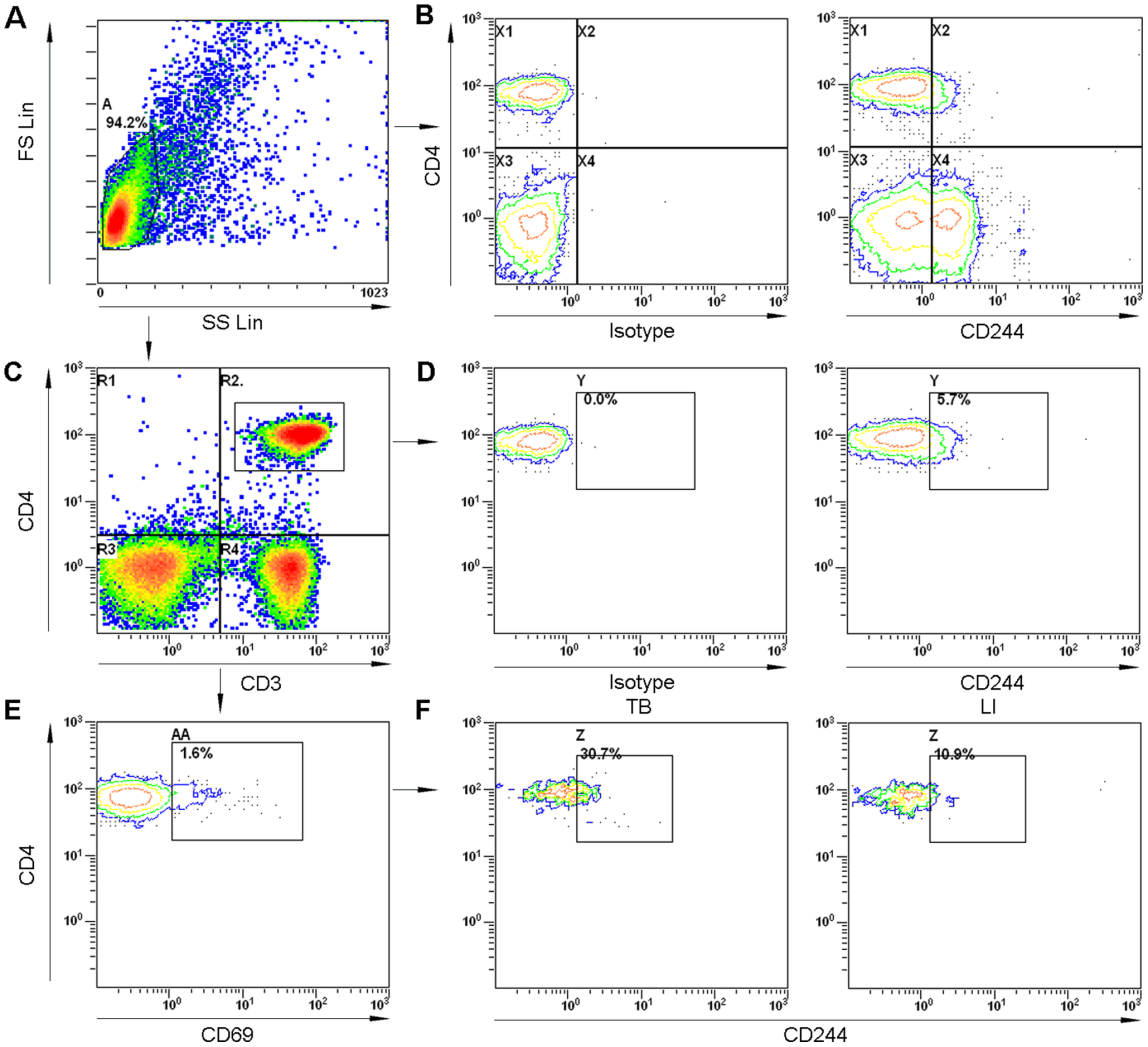
Representative flow cytometric plots showing gating strategy and expression of CD244/2B4. (A) Viable lymphocytes in PBMCs were gated based on characteristic forward and side scatter profiles. (B) The gate depicting CD4/CD244 expression was defined by both control isotype staining and CD244 expression on CD3^−^CD4^−^ cells that showed clearly two cell subsets with positive and negative staining of CD244. (C and D) CD4^+^ T cells were defined as CD3^+^CD4^+^ cells (C) and CD244/2B4 expression on total CD4^+^ T cells was shown (D). (E and F) Antigen-responsive CD4**^+^** T cells were identified by appearance of activation marker CD69 in the CD3^+^CD4^+^ cell population (E) and CD244/2B4 expression on *M. tuberculosis* antigen-responsive CD4^+^ T cells was shown (F). Isotype: isotype antibody control; LI: latent TB infection; TB: active TB patients.

**Figure 2:**
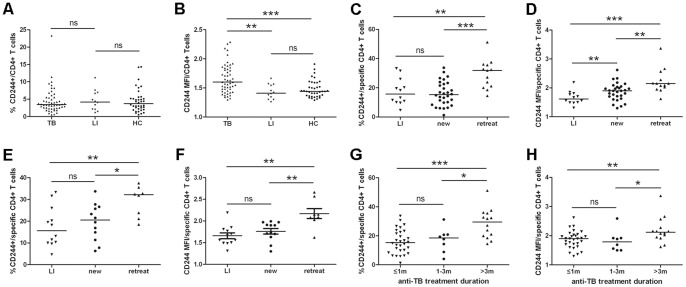
The expression of CD244/2B4 on CD4^+^ T cells in active pulmonary TB patients, latent infection individuals and healthy controls. (A) The frequencies of CD244/2B4-expressing total CD4^+^ T cells were similar among active TB patients, latent infection individuals and healthy controls. (B) Mean fluorescent intensity (MFI) of CD244/2B4 expression on total CD4^+^ T cells was significantly higher in active TB patients than in latent infection individuals and healthy controls. (C) The frequencies of CD244/2B4-expressing antigen-responsive CD4^+^ T cells increased significantly in retreatment active TB patients as compared with latent infection individuals and new active TB patients. (D) MFI of CD244/2B4 expression on antigen-responsive CD4^+^ T cells was significantly higher in retreatment active TB patients as compared with latent infection individuals and new active TB patients. New TB patients had significantly elevated expression of CD244/2B4 as compared with latent infection individuals. (E and F) When only *M. tuberculosis* culture-positive TB cases were selected for analysis, retreatment TB patients also had higher frequencies of CD244/2B4-expressing antigen-responsive CD4^+^ T cells (F) and elevated expression of CD244/2B4 as compared with latent infection individuals and new TB patients (G). (G and H) TB patients with more than 3 months of treatment, which contained all of the retreatment cases, had higher frequencies of CD244/2B4-expressing antigen-responsive CD4^+^ T cells (G) and elevated expression of CD244/2B4 as compared with those who were treated less than 1 month or between 1 to 3 months (H). HC: healthy controls; new: new TB patients; retreat: retreatment TB patients; ≤1 m: less than 1 month of treatment; 1–3 m: between 1 to 3 month of treatment; ≥3 m: more than 3 month of treatment. Mann-Whitney test was used for statistical analysis between groups. *: p<0.05; **: p<0.01; ***: p<0.001.

Since CD4^+^ T cells play critical role in protective immunity to TB, we studied the expression and role of CD244/2B4 on CD4^+^ T cells. PBMCs from patients with pulmonary TB and healthy controls were stained with fluorescence-labeled CD3, CD4 and CD244/2B4 antibodies and analyzed by flow cytometry (Fig. 1). The gate depicting CD4/CD244 expression was defined by both isotype staining and CD244 expression on CD3^−^CD4^−^ cells which showed clearly two cell subsets with positive and negative staining of CD244 (Fig. 1A to 1D). The frequencies of CD244/2B4-expressing total CD4^+^ T cells were similar among active TB patients (n = 52), latent infection individuals (n = 12) and healthy controls (n = 38) (Fig. 2A), while the expression levels of CD244/2B4 on total peripheral CD4^+^ T cells were significantly higher in active TB patients than in latent infection individuals (p = 0.0025) and healthy controls (p = 0.0003) (Fig. 2B).

### Association of CD244/2B4 expression on *M. tuberculosis* antigen-responsive CD4^+^ T cells with disease outcome of TB patients

To further investigate the role of CD244/2B4 on *M. tuberculosis* antigen-responsive CD4^+^ T cells, PBMCs from patients with active TB patients and latent TB infection were stimulated with *M. tuberculosis* antigens overnight. Antigen-responsive CD4**^+^** T cells were identified by appearance of activation marker CD69 in the CD3^+^CD4^+^ cell population (Fig. 1E) [29], [30]. In the cohort of TB patients, 30 were defined as new cases and 13 retreatment TB patients (Table 1). The frequencies of CD244/2B4-expressing antigen-responsive CD4^+^ T cells increased significantly in retreatment active TB patients as compared with latent infection individuals (p = 0.0051) and new active TB patients (p = 0.0004) (Fig. 1F and 2C), while the difference between new active TB patients and latent infection individuals was not significant (Fig. 2C). Mean fluorescent intensity (MFI) of CD244/2B4 expression on antigen-responsive CD4^+^ T cells was also significantly higher in retreatment active TB patients as compared with latent infection individuals (p = 0.0006) and new active TB patients (p = 0.0029) (Fig. 1F and 2D). New TB patients had significantly elevated expression of CD244/2B4 as compared with latent infection individuals (p = 0.0029) (Fig. 1F and 2D).

When only *M. tuberculosis* culture-positive TB cases were selected for analysis, retreatment TB patients were found to have higher frequencies of CD244/2B4-expressing antigen-responsive CD4^+^ T cells than latent infection individuals (p = 0.0098) and new TB patients (p = 0.0339) as well (Fig. 2E). The expression of CD244 on antigen-responsive CD4^+^ T cells was elevated in retreatment TB patients compared with latent infection individuals (p = 0.0048) and new TB patients (p = 0.0038) (Fig. 2F).

To determine whether anti-TB chemotherapy affects CD244 expression on CD4 T cells, TB patients were divided into three groups according to treatment duration. CD244 expression on antigen-responsive CD4^+^ T cells was significantly higher in patients with more than 3 months of treatment that contains all of the retreatment cases than those treated less than 1 month or between 1 to 3 months (Fig. 2G and 2H).

Taken together, these results indicate that active TB patients have significantly elevated expression of CD244/2B4 on *M. tuberculosis* antigen-responsive CD4^+^ T cells.

### Functional analysis of CD244/2B4- expressing antigen-responsive CD4^+^ T cells

**Figure 3:**
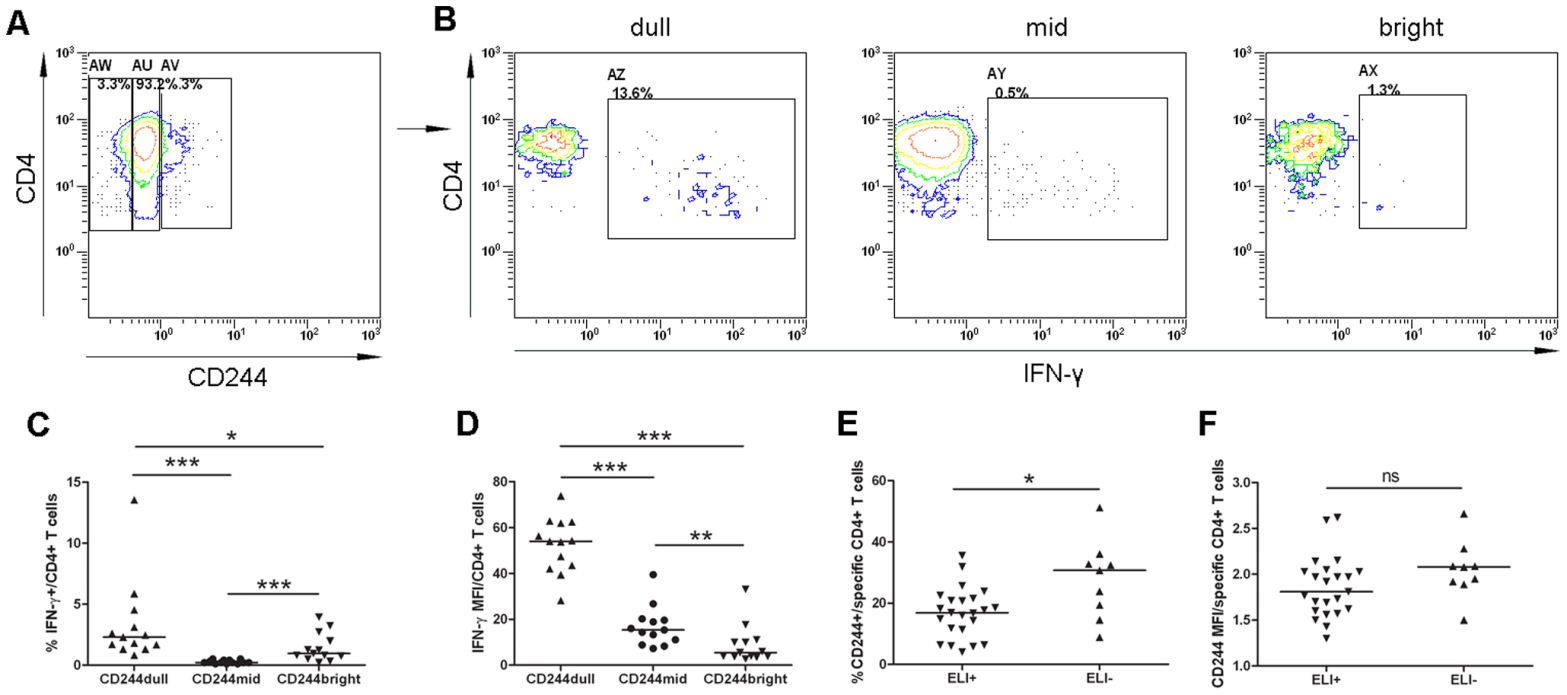
Influence of CD244/2B4 expression on IFN-γ production. (A and B) Representative flow cytometric plots showing gating strategy of CD244/2B4-dull (AW) –middle (AU) and -bright (AV) CD4^+^ T cells, and Intracellular IFN-γ production in CD244/2B4-dull (AZ), -middle (AY) and -bright (AX) CD4^+^ T cells. (C) CD244/2B4-bright CD4^+^ T cells contained significantly lower ratio of IFN-γ-producing CD4^+^ cells, compared with CD244/2B4-dull CD4^+^ T cells. (D) CD244/2B4-bright CD4^+^ T cells had greatly reduced expression of IFN-γ, compared with CD244/2B4-dull and –middle CD4^+^ T cells. (E and F) T-SPOT.TB-negative TB patients had higher frequencies of CD244/2B4-expressing cells than T-SPOT.TB-positive TB patients. ELI+: ELISPOT-positive TB patients; ELI-: ELISPOT-negative TB patients. Mann-Whitney test was used for statistical analysis between groups. *: p<0.05; ***: p<0.001.

To understand functional relevance of elevated CD244/2B4 expression on CD4^+^ T cells, PBMCs from TB patients were stimulated with *M. tuberculosis* strain H37Rv lysates, and the production of effector molecule IFN-γ in CD4^+^ T cells were examined by flow cytometry (Fig. 3A and 3B). CD244/2B4-bright CD4^+^ T cells contained significantly lower ratio of IFN-γ-producing CD4^+^ cells (p = 0.0224) than CD244/2B4-dull CD4^+^ T cells (Fig. 3B and 3C), and had greatly reduced expression of IFN-γ) compared with CD244/2B4-dull (p<0.0001) and -middle CD4^+^ T cells (p = 0.0259) (Fig. 3B and 3D).

Comparison of CD244 expression on antigen-responsive CD4^+^ T cells according to ELISPOT results showed that T-SPOT.TB-negative TB patients had higher frequencies of CD244/2B4-expressing cells than T-SPOT.TB-positive TB patients (Fig. 2E and F).

These results suggest that CD244/2B4 expression on antigen-specific CD4^+^ T cells influences IFN-γ production.

### Activation of CD244/2B4 signaling results in significantly decreased IFN-γ production

**Figure 4:**
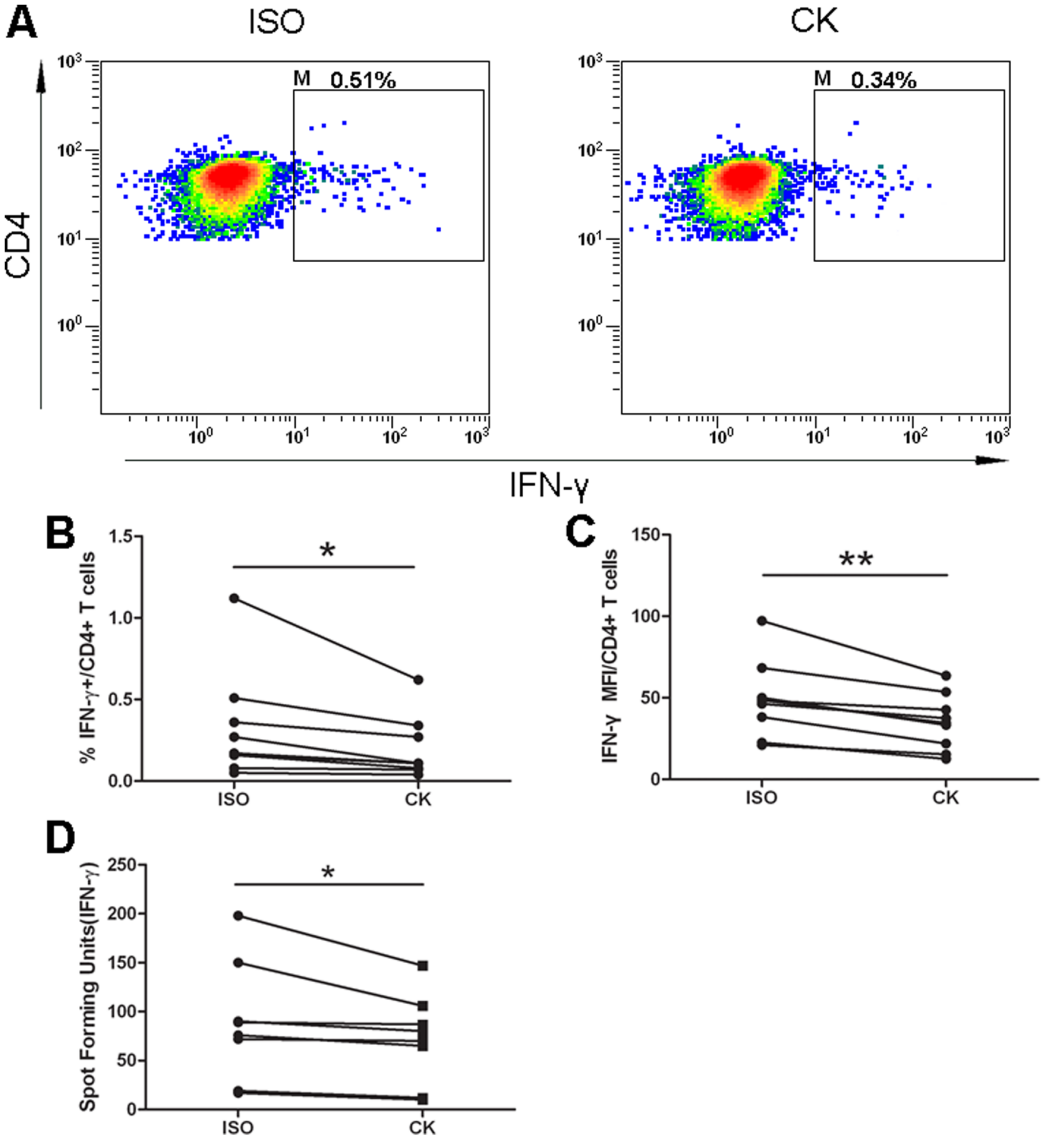
Activation of CD244/2B4 signaling resulted in significantly decreased IFN-γ production. (A) Representative flow cytometric plots showing IFN-γ production in CD3^+^CD4^+^ T cells incubated with isotype control antibody (ISO) or with anti-CD244/2B4 antibody clone C1.7 (CK). (B to D) Activation of CD244/2B4 signaling by cross-linking led to significantly lower frequencies of IFN-γ-producing CD4^+^ T cells (B), decreased expression of IFN-γ as determined by flow cytometry (C), and reduced numbers of spot forming units in ELISPOT assay (D). Paired t-test was used for statistical analysis. *: p<0.05; **: p<0.01.

Anti-CD244/2B4 antibody clone C1.7 is known to be a cross-linking antibody that activates CD244/2B4 receptor signaling [31]. To study the role of CD244/2B4 receptor signaling, anti-2B4 antibody clone C1.7 was added to culture medium during *M. tuberculosis* antigen stimulation of human PBMCs from patients with active TB. Activation of CD244/2B4 signaling by cross-linking led to significantly lower frequencies of IFN-γ-producing CD4^+^ T cells (p = 0.0377) (Fig. 4A and4B), decreased production of IFN-γ (p = 0.0011) (Fig. 4A and 4C) and also reduced numbers of spot forming units (SFU) in ELISPOT assay (p = 0.0442) (Fig. 4D). The result indicates that CD244/2B4 plays an inhibitory role on CD4^+^ T cells in patients with active TB.

### Rescue of IFN-γ production following CD244/2B4 receptor blocking

**Figure 5:**
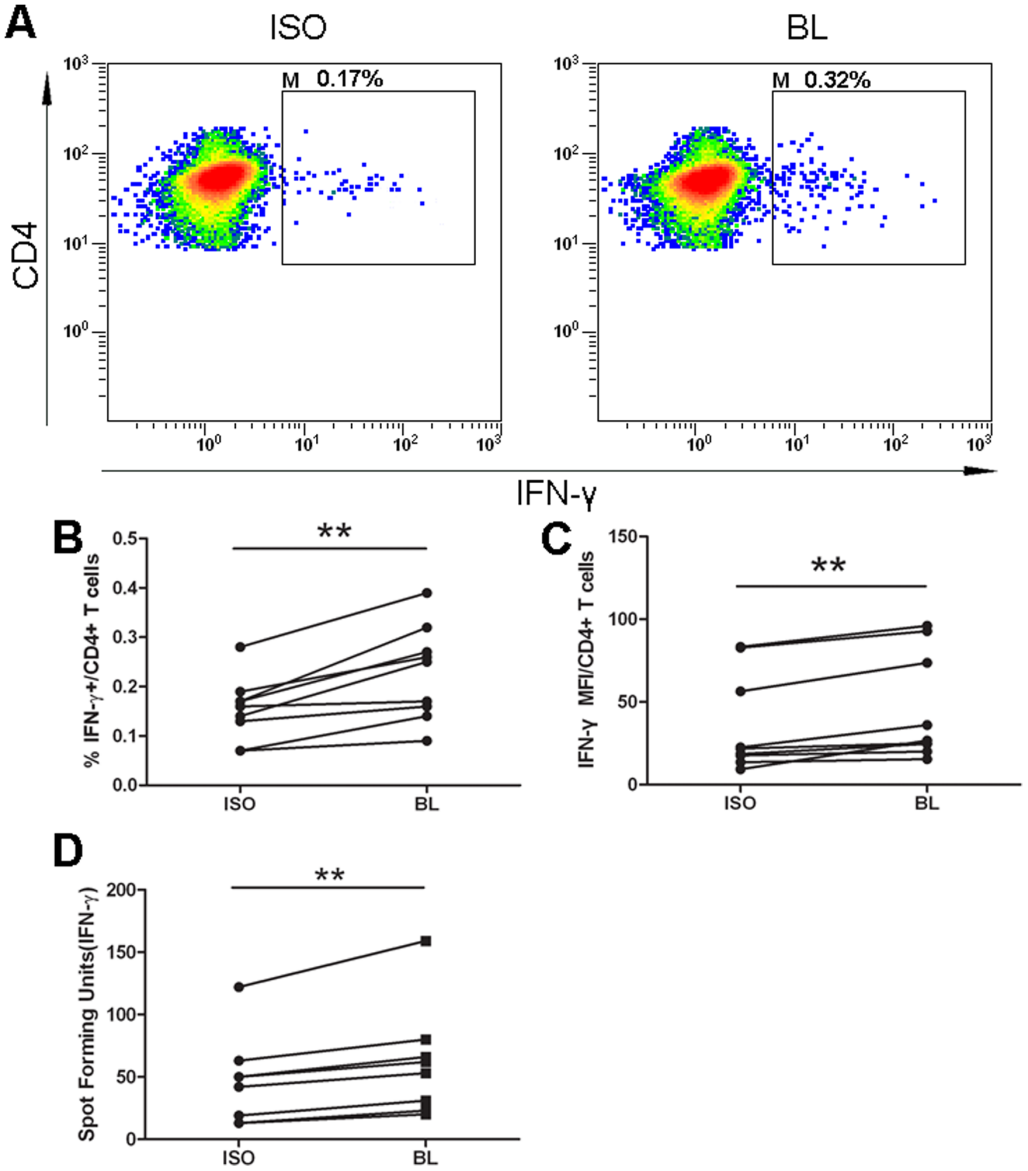
Rescue of IFN-γ production following CD244/2B4 receptor blocking. (A) Representative flow cytometric plots showing IFN-γ production in stimulated CD3^+^CD4^+^ T cells when incubated with isotype control antibody (ISO) or blocking anti-CD244/2B4 antibody clone eBioPP35 (BL). (B to D) Blocking of CD244/2B4 signaling resulted in significantly higher frequencies of IFN-γ-producing CD4^+^ T cells (B), increased level of IFN-γ production in CD3^+^CD4^+^ T cells as determined by flow cytometry (C), and elevated numbers of spot forming units in ELISPOT assay (D), as compared with those incubated with isotype control antibody. Paired t-test was used for statistical analysis. **: p<0.01.

To determine whether blocking CD244/2B4 signaling pathway could rescue functional defects of T cells, a CD244/2B4 receptor blocking antibody was used. Previous study found that anti-human CD244/2B4 monoclonal antibody clone eBioPP35 recognizes different epitope from clone C1.7 and is a blocking antibody of CD244/2B4 receptor [26]. Addition of anti-human CD244/2B4 monoclonal antibody clone eBioPP35 to the culture medium of human PBMCs from patients with active TB during *M. tuberculosis* antigen stimulation resulted in significantly higher frequencies of IFN-γ-producing CD4^+^ T cells (p = 0.0015) (Fig. 5A and 5B) and increased production of IFN-γ (p = 0.0018) (Fig. 5A and 5C) and also elevated numbers of SFU in ELISPOT assay (p = 0.0024) (Fig. 5D), compared with isotype antibody controls. This result suggests that decreased production of IFN-γ could be rescued by manipulation of CD244/2B4 receptor signaling.

## Discussion

The influence of CD244/2B4 on CD4^+^ T cell immunity has not been reported so far. Since CD4^+^ T cells play critical role in protective immunity against *M. tuberculosis* infection, we investigated the role of CD244/2B4 on antigen-specific CD4^+^ T cells. We found that pulmonary TB patients had significantly elevated CD244/2B4 expression on *M. tuberculosis* antigen-specific CD4^+^ T cells compared with latent infection individuals.

The role of CD244/2B4 on CD8 T cells in infectious diseases has been reported recently. CD244/2B4 was highly unregulated on exhausted CD8^+^ T cell during chronic lymphocytic choriomeningitis virus (LCMV) infection [24], [25]. In chronic hepatitis B virus (HBV) infection, virus-specific CD8^+^ T cells expressed higher levels of CD244/2B4 both in the peripheral blood and liver. The blockage of the CD244/2B4 signaling pathway by antibodies directed against either CD244/2B4 or its ligand CD48 resulted in an increased virus-specific proliferation and cytotoxicity, suggesting an inhibitory function of the receptor [23]. During chronic LCMV infection, CD244/2B4 is most highly up-regulated on the secondary CD8^+^ effector T cells and is associated with loss of CD8^+^ memory T cells [27]. In patients with acute and chronic hepatitis C, CD244/2B4 is found to be present on virus-specific CD8^+^ T cells and is a marker for CD8^+^ T cell dysfunction. CD244/2B4 receptor cross-linking can lead to both inhibition and activation of HCV-specific CD8^+^ T cell function, depending on expression levels of CD244/2B4 [26]. In HTLV-I-infected patients, CD244 expression was significantly higher on CD8^+^ T cells. Blockade of CD244/2B4 receptor signaling inhibits degranulation and IFN-γ production in CD8^+^ T cells of patients with HTLV-I-associated myelopathy/tropical spastic paraparesis (HAM/TSP), suggesting that CD244/2B4 might play roles in promoting inflammatory neurological disease [32].

CD4^+^ T cells secreting IFN-γ and other cytokines play a critical role in protective immunity against TB. IFN-γ can activate macrophage to kill *M. tuberculosis* by inducing expression of nitric oxide synthase and production of reactive nitrogen intermediates (RNI) [10], [33]. Immune therapy with anti-TNF antibody impairs antimicrobial activity of CD8^+^ T cells against *M. tuberculosis* and leads to increased risk of active TB [34]. Polyfunctional CD4^+^ T cells that simultaneously express IFN-γ, TNF-α and IL-2 may be associated with control of *M. tuberculosis* infection [35], [36], [37]. We found that CD244/2B4-bright CD4^+^ T cell subset had significantly reduced production of IFN-γ, suggesting that CD244/2B4-expressing CD4^+^ T cells may have functional defects. Activation of CD244/2B4 signaling pathway by cross-linking can leads to inhibition of IFN-γ production. Therefore, CD244/2B4 may play an inhibitory role on *M. tuberculosis* antigen-specific CD4^+^ T cell function.

In the cohort of TB patients recruited in the study, 13 had failed treatment. Our study showed that TB patients with failed treatment had significantly elevated expression of CD244/2B4 on antigen-specific CD4^+^ T cells compared with new TB patients. Previous studies found that persistent antigen stimulation of T cells during chronic infection can results in T cell exhaustion that fails to produce effector cytokines upon antigen stimulation, and CD244/2B4 was found to be highly unregulated on dysfunctional CD8^+^ T cell [24], [25]. Persistent existence of *M. tuberculosis*, especially in TB patients with failed treatment, may leads to T cell dysfunction with reduced bactericidal capacity. Inhibitory receptors such as CD244/2B4, Tim-3 and PD-1 could be used as biomarkers for T cell dysfunction in TB patients, however, we did not look at the expression of PD-1 in the same patients and this was a limitation of the study.

In summary, our study demonstrated that CD244/2B4 expression on *M. tuberculosis* antigen-responsive CD4^+^ T cells was significantly higher in active TB patients than in latent TB infection individuals. CD244/2B4 expression was negatively correlated with IFN-γ production and it has an inhibitory role on *M. tuberculosis* antigen-specific CD4^+^ T cell function.
